# A Review of Resistance Mechanisms of Synthetic Insecticides and Botanicals, Phytochemicals, and Essential Oils as Alternative Larvicidal Agents Against Mosquitoes

**DOI:** 10.3389/fphys.2019.01591

**Published:** 2020-02-25

**Authors:** Sengottayan Senthil-Nathan

**Affiliations:** Division of Biopesticides and Environmental Toxicology, Sri Paramakalyani Centre for Excellence in Environmental Sciences, Manonmaniam Sundaranar University, Tirunelveli, India

**Keywords:** biopesticide, vector, secondary metabolites, phytochemical, physiology, enzyme, toxicity

## Abstract

Mosquitoes are a serious threat to the society, acting as vector to several dreadful diseases. Mosquito management programes profoundly depend on the routine of chemical insecticides that subsequently lead to the expansion of resistance midst the vectors, along with other problems such as environmental pollution, bio magnification, and adversely affecting the quality of public and animal health, worldwide. The worldwide risk of insect vector transmitted diseases, with their associated illness and mortality, emphasizes the need for effective mosquitocides. Hence there is an immediate necessity to develop new eco-friendly pesticides. As a result, numerous investigators have worked on the development of eco-friendly effective mosquitocidal compounds of plant origin. These products have a cumulative advantage of being cost-effective, environmentally benign, biodegradable, and safe to non-target organisms. This review aims at describing the current state of research on behavioral, physiological, and biochemical effects of plant derived compounds with larvicidal effects on mosquitoes. The mode of physiological and biochemical action of known compounds derived from various plant families as well as the potential of plant secondary metabolites, plant extracts, and also the essential oils (EO), as mosquitocidal agents are discussed. This review clearly indicates that the application of vegetal-based compounds as mosquito control proxies can serve as alternative biocontrol methods in mosquito management programes.

## Introduction

Vector borne diseases account for more than seven million deaths annually (World Health Organization [WHO], 2017), among which mosquito borne diseases are the most threatening due to their wide spread occurrence, consequently featuring a higher frequency of disease transmission (Lounibos, 2002; Tyagi et al., 2015). Among different mosquito families, Culicidae is a large family (3,300 Service species-41 genera) comprising *Toxorhynchitinae*, *Anophelinae* (anophelines), and also *Culicinae* (culicines) sub-families (Service, 1996; Senthil-Nathan et al., 2005b). Among the 31 genera, *Anopheles*, *Culex*, and *Aedes* are the most detrimental. *Anopheles* species, are carriers of major life-threatening diseases (malaria and filariasis-transmitting agents, such as *Wuchereria bancrofti*, *Brugia malayi*, and *Brugia timori*) and also of a few arboviruses (Kalaivani et al., 2012; Benelli et al., 2018; Thanigaivel et al., 2019; Vasantha-Srinivasan et al., 2019).

The discovery of DDT’s insecticidal properties in late 1930s/beginning of 1940s and the following progress of organochlorine invention and organophosphate insecticides concealed biological pesticide merchandise-research since the responses to mosquito regulation were supposed to have remained established (Shaalan et al., 2005; Senthil-Nathan et al., 2006a, b). The ranges of many of the mosquito species were not limited and keep expanding, thereby up surging the rates of disease incidence. Until recently, the use of several of the earlier synthetic-insecticides, such as permethrin and malathion, along with other organophosphates in vector control programes has been partial. This is due to absence of unique-insecticides, expense of synthetic-insecticides, apprehension for ecological sustainability, damaging influence on human health, besides further non-target populations, their persistent nature, greater amount of “biological magnification” through ecosystem and also the development of insecticide resistance (Ghosh et al., 2012). The emergence of DDT resistance in *Aedes* species (*Ae. tritaeniorhynchus* and *Ae. sollicitans*) lead to numerous drawbacks in mosquito control programs (Brown, 1986). Several categories of Mosquitocides are being implemented in malaria control programs (BHC, organophosphorus, carbamate, and pyrethroid). The ability of mosquitoes to evade the insecticidal action of these synthetic compounds are attributed to the increase in the rate of synthesis of detoxifying enzymes such as monoxygenases (MFOs), glutathione-*S*-transferases (GST) and carboxyl-cholinesterase (CCE). MFOs are often associated with metabolic resistance to pyrethroids, such as permethrin, while GSTs are usually associated with organochloride resistance such as DDT. Resistance to pyrethroids, organophosphates and carbamates, such as bendiocarb are incurred by the magnification of CCE activity (Hemingway and Ranson, 2000). Added insecticides, benzylphenyl urea and the larvicide, *Bacillus thuringiensis israelensis* (Bti), have partial use against mosquitoes. Unpredicted natural or anthropogenic associated ecological variations that modify the original habitats severely affect the vector biology thereby positively influencing their existence and disease incidence, thus constraining the frame-work of mosquito control strategies.

## Biological Management of Mosquitoes

Several phytochemicals from several plant families are identified with larvicidal activities against different mosquito species (Table 1). Plant extracts with their augmented phytochemical elements have a recognized potential as a substitute to conventional mosquito control agents (Sukumar et al., 1991; Tripathi et al., 2009; Tehri and Singh, 2015). The main strategy for mosquito control deals with the restriction of the vector population. As a promising biocontrol agent, the compounds from the plants of the family *Meliaceae* such as neem *Azadirachta indica* A. Juss (Senthil-Nathan et al., 2005b; Senthil-Nathan, 2013), Indian white cedar, *Dysoxylum malabaricum* Bedd. (Senthil-Nathan et al., 2006a), *D. beddomei* and chinaberry tree, *Melia azedarach* L. (Senthil-Nathan et al., 2006b) were effective against *An. stephensi* (Senthil-Nathan et al., 2008). “Secondary metabolites” from *Eucalyptus tereticornis* Sm. (forest redgum, *Myrtaceae*) exhibited effective mosquitocidal activities against *An. stephensi* as reported by Senthil-Nathan (2007). Also, the crude metabolic extracts of *Acanthospermum hispidum* leaves were active against *An. stephensi*, *Ae. Aegypti*, as well as *Cx. quinquefasciatus* as reported by Vivekanandhan et al. (2018a, b). A study conducted on testing the mosquitocidal activity of *Justicia adhatoda* L. (Acanthaceae) leaf extracts revealed the potential of natural larvicidal agent against *Ae. Aegypti* (Thanigaivel et al., 2012, 2017a,b).

**TABLE 1:** Phytochemicals identified from the specific plant families and their larvicidal activity on the mosquito species.

**Family and plant species**	**Major constituents**	**Mosquito species**	**References**
**Acanthaceae**			
*Andrographis paniculata*	Andrographolide	*Aedes aegypti*	Edwin et al., 2016
**Alangiaceae**			
*Alangium salvifolium*	Asarinin, sesamin and (+)-xanthoxylol-γ,γ-dimethylallylether, Hexadecanoicacid,1 hydroxymethyl-1,2-ethanediyl ester	*Aedes aegypti*	Thanigaivel et al., 2017a
**Amaranthaceae**			
*Chenopodium ambrosioides*	α-Terpineol	*Aedes aegypti*	Leyva et al., 2009b
**Amaryllidaceae**			
*Alium macrostemon*	Methyl propyl disulfide; mimethyl trisulfide	*Aedes albopictus*	Liu et al., 2014a
*Alium monanthum*	Dimethyl trisulfide; dimethyl tetrasulfide	*Aedes aegypti*	Moon, 2011
**Anacardiaceae**			
*Pistacia terebinthus*	α-Pinene; cyclopentane	*Culex quinquefasciatus*	Cetin et al., 2011
*Spondias purpurea*	Caryophyllene oxide and α-cadinol	*Aedes aegypti*	Lima et al., 2011
**Annonaceae**			
*Cananga odorata*	Benzyl acetate, linalool, methyl benzoate	*Aedes aegypti*	Vera et al., 2014
*Guatteria blepharophylla*	Caryophyllene oxide	*Aedes aegypti*	Aciole et al., 2011
*Guatteria friesiana*	β-Eudesmol	*Aedes aegypti*	Aciole et al., 2011
*Guatteria hispida*	β-Pinene and α-pinene	*Aedes aegypti*	Aciole et al., 2011
*Rollinia leptopetala*	Spathulenol	*Aedes aegypti*	Feitosa et al., 2009
**Apiaceae**			
*Angelica purpuraefolia*	4’-Chloro-4,4-dimethyl-3-(1-imidazolyl)-valerophenone, 1-Dodecanol,	*Aedes aegypti*	Nagella et al., 2012
*Anethum graveolens*	Limonene, carvone	*Aedes albopictus*	Seo et al., 2015
*Apium graveolens*	R-+-Limonene	*Aedes aegypti*	Pitasawat et al., 2007
	Limonene, carvone	*Aedes albopictus*	Seo et al., 2015
*Bupleurum fruticosum*	α-Pinene; β-pinene	*Culex pipiens*	Evergetis et al., 2009
*Carum carvi*	Carvone	*Aedes aegypti*	Pitasawat et al., 2007
*Conopodium capillifolium*	α-Pinene; sabinene	*Aedes aegypti*	Evergetis et al., 2009
*Coriandrum sativum*	Linalool, 2,6-octadien-1-ol, 3,7- dimethyl-, acetate, E-	*Aedes aegypti*	Nagella et al., 2012
*Cuminum cyminum*	ρ-cymene, β-pinene, cuminaldehyde	*Aedes albopictus*	Seo et al., 2015
*Daucus carota*	Carotol	*Aedes albopictus*	Seo et al., 2015
*Elaeoselinum asclepium*	α-Pinene; sabinene	*Aedes aegypti*	Evergetis et al., 2009
*Foeniculum vulgare*	*trans*-Anethole, Limonene	*Aedes aegypti*	Rocha et al., 2015
*Heracleum pastinacifolium*	Octyl acetate, Hexyl	*Aedes aegypti*	Tabanca et al., 2012a
*Ligusticum chuanxiong*	octadecenoic acids	*Aedes aegypti, Culex quinquefasciatus*	Evergetis et al., 2009
*Oenanthe pimpinelloides*	γ-Terpinene; o-cymene	*Aedes aegypti*	Pavela, 2015
*Pimpinella anisum*	Trans-anethole, α-Pinene; sabinene, β-phellandrene	*Aedes aegypti*	Pavela, 2015
*Petroselinum crispum*	β-phellandrene,myristicin, α & β-pinene, myrcene	*Anopheles culicifacies*	Evergetis et al., 2012
*Pe. Sativum*	Myristicin,1,8-cineole, 1,3,8-p-menthatriene	*Aedes albopictus*	Seo et al., 2015
*Trachyspermum ammi*	Thymol	*Anopheles stephensi*	Pandey et al., 2009
	ρ-Cymene, γ-Terpinene	*Aedes albopictus*	Seo et al., 2015
**Apocynaceae**			
*Cionura erecta L.*	Edren-9-one, alpha cadinol, eugenol and alpha muurolene	*Anopheles stephensi*	Mozaffari et al., 2014
**Araliaceae**			
*Dendropanax morbifera*	γ-Elemene	*Aedes aegypti*	Chung et al., 2009
***Aristolochiaceae***			
*Aristolochia indica*	Aristolochic acid I and II	*Aedes aegypti*	Pradeepa et al., 2015
*Asarum heterotropoides*	Methyleugenol and safrole	*Aedes aegypti*	Perumalsamy et al., 2009
**Asteraceae**			
*Achillea millefolium*	Eucalyptol, β-pinene, borneol, sabinene, camphene	*Aedes albopictus*	Conti et al., 2010
*Artemisia absinthium*	(Z)-β-ocimene, (E)-β-farnesene (Z)-en-yn-dicycloether	*Aedes aegypti, Culex quinquefasciatus, Anopheles stephensi*	Govindarajan and Benelli, 2016
*Ar. dracunculus*	Hexanal, isovaleric acid, (Z)-3-hexenol,	*Anopheles stephensi*	Pour et al., 2016
	Hexadecanol		
*Artemisia vulgaris*	Camphor, Linalool, terpenen-4-ol, a-and bthujone, b-pinene	*Aedes aegypti*	Bora and Sharma, 2011
	camphor, alpha-thujone, betacaryophyllene, gammamuurolene, camphene		
*Artemisia vulgaris*	Myrcene, limonene, cineol	*Aedes aegypti*	Sujatha et al., 2013
*Ar. Nilagirica*	Capillin	*Aedes aegypti, Aedes albopictus*	Bora and Sharma, 2011
*Blumea densiflora*	Borneol, germacrene D, β-caryophyllene, γ-terpinene, sabinene, β-bisabolene	*Anopheles anthropophagus*	Zhu and Tian, 2011
*Blumea mollis*	Linalool, γ-elemene, copaene, estragole, Allo-ocimene, γ-terpinene Alloaromadendrene	*Culex quinquefasciatus*	Senthilkumar et al., 2008
*Chamaemelum nobile*	α-pinene	*Aedes aegypti, Culex quinquefasciatus*	Amer and Mehlhorn, 2006
*Chrysanthemum indicum*	verbenol, 1,8-cineole, α-pinene, camphor, borneol, bornyl acetate	*Aedes aegypti*	Shunying et al., 2005
		*Aedes aegypti*	Wu et al., 2010
*Eupatorium betonicaeforme*	β-Caryophyllene	*Aedes aegypti*	Albuquerque et al., 2004
*Matricaria recutita*	α-bisabolol	*Aedes aegypti*	Heuskin et al., 2009
*Pectis oligocephala*	*p*-Cymene and thymol	*Aedes aegypti*	Albuquerque et al., 2007
*Tagetes erecta*	Piperitone	*Aedes aegypti*	Marques et al., 2011
*Tagetes filifolia*	*trans*-Anethole	*Aedes aegypti*	Ruiz et al., 2011
*Tagetes lucida*	Methyl chavicol	*Aedes aegypti*	Vera et al., 2014
*Tagetes minuta*	Trans-ocimenone	*Aedes aegypti*	Ruiz et al., 2011
*Tagetes minuta*	5E-ocimenone	*Aedes aegypti*	Maradufu et al., 1978
*Tagetes patula*	Limonene and terp	*Aedes aegypti*	Dharmagadda et al., 2005
**Bignoniaceae**			
*Cybistax antisyphilitica*	quinone	*Aedes aegypti*	Rodrigues et al., 2005
**Boraginaceae**			
*Auxemma glazioviana*	α-Bisabolol, α-cadinol, and T-muurolol	*Aedes aegypti*	Costa et al., 2004
*Cordia curassavica*	Cordiaquinones J and K	*Aedes aegypti*	Ioset et al., 2000
	α-Pinene	*Aedes aegypti*	Santos et al., 2006
*Cordia leucomalloides*	δ-Cadinene and E- caryophyllene	*Aedes aegypti*	Santos et al., 2006
**Cucurbiataceae**			
*Bryonopsis laciniosa*	Goniothalamin	*Culex pipiens*	Kabir et al., 2003
**Cupressaceae**			
*Callitris glaucophylla*	Guaiol & citronellic acid	*Aedes aegypti*	Shaalan et al., 2006
*Chamaecyparis formosensis*	Myrtenol	*Aedes aegypti*	Kuo et al., 2007
*Cryptomeria japonica*	16-Kaurene and elemol	*Aedes aegypti, Aedes albopictus*	Cheng et al., 2009c
*Cunninghamia konishii*	Cedrol, α-Pinene	*Aedes aegypti*	Cheng et al., 2013
*Cupressus arizonica var. glabra*	α-Pinene & epi-zonarene	*Aedes aegypti*	Ali et al., 2013
*Cupressus arizonica*	Limonene, umbellulone α-pinene	*Anopheles stephensi*	Sedaghat et al., 2011
*Cupressus benthamii*	Limonene; umbellulone	*Aedes albopictus*	Giatropoulos et al., 2013
*Cupressus macrocarpa*	Sabinene; α-Pinene; terpinen-4-ol	*Aedes albopictus*	Giatropoulos et al., 2013
*Cupressus sempervirens*	α-Pinene; δ-3-carene	*Aedes albopictus*	Giatropoulos et al., 2013
*Cupressus torulosa*	α-Pinene; δ-3-carene	*Aedes albopictus*	Giatropoulos et al., 2013
*Chamaecyparis*	Myrtenol; myrtenal	*Aedes aegypti, Aedes aegypti*	Kuo et al., 2007
*formosensis*	Limonene; oplopanonyl acetate; beyerene	*Aedes albopictus*	Giatropoulos et al., 2013
*Chamaecyparis lawsoniana*	α-Pinene; sabinene; δ-3-carene	*Culex pipiens*	Vourlioti-Arapi et al., 2012
*Juniperus communis* ssp.			
*Hemisphaerica*	α-Pinene; limonene	*Culex pipiens*	Vourlioti-Arapi et al., 2012
*Juniperus drupacea*	Sabinene; 4-methyl-1-1-methylethyl-3-cyclohexen-1-ol	*Culex pipiens*	Vourlioti-Arapi et al., 2012
*Juniperus foetidissima*	Myrcene; germacrene-D; α-Pinene	*Culex pipiens*	Vourlioti-Arapi et al., 2012
*Juniperus oxycedrus L.* ssp.			
*oxycedrus*	α –pinene	*Culex pipiens*	Vourlioti-Arapi et al., 2012
*Juniperus oxycedrus L.*			
*subsp. Macrocarpa*	α-Pinene; δ-3-carene; β-phellandrene; α-terpinyl acetate	*Aedes albopictus*	Giatropoulos et al., 2013
*Juniperus phoenicea*			
*Tetraclinis articulate*	α-Pinene; bornyl acetate	*Aedes albopictus*	Giatropoulos et al., 2013
**Dioncophyllaceae**			
*Triphyophyllum peltatum*	dioncophylline A	*Anopheles stephensi*	François et al., 1996
**Euphorbiaceae**			
*Croton nepetaefolius*	Methyleugenol	*Aedes aegypti*	Morais et al., 2006
*Croton regelianus*	Ascaridole & *p*-Cymene	*Aedes aegypti*	Torres et al., 2008
*Croton zehntneri*	*E*-anethole, *p*-anisaldehyde	*Aedes aegypti*	Morais et al., 2006
**Fabaceae**			
*Copaifera multijuga*	β-caryophyllene	*Anopheles darling, Aedes aegypti*	Trindade et al., 2013
*Hymenaea courbaril*	α-Copaene, spathulenol	*Aedes aegypti*	Aguiar et al., 2010
	Germacrene D and β-caryophyllene		
*Myroxylon pereirae*	Benzyl benzoate	*Aedes aegypti*	Yenesew et al., 2003
*Millettia dura*	Rotenoids, deguelin and tephrosin caryophyllene oxide; phenol,4-3,7-dimethyl-3-ethenylocta-1,6-dienyl; caryophyllene	*Culex quinquefasciatus*	Dua et al., 2013
*Psoralea corylifolia*	Citronellol	*Aedes aegypti*	Benelli et al., 2017
**Geraniaceae**		*Culex quinquefasciatus*	Cavalcanti et al., 2004
*Pelargonium graveolens*	Neral; geranial	*Culex quinquefasciatus*	
**Gramineae**	α-Pinene	*Aedes aegypti*	Cetin et al., 2011
*Cymbopogon citratus*	Thymol	*Culex pipiens*	Govindarajan et al., 2013
**Hypericaceae**			
*Hypericum scabrum*	Δ-3-carene, 1,8-cineole, β-caryophyllene, bicyclogermacrene	*Culex tritaeniorhynchus, Aedes albopictus, and Anopheles subpictus*	Araújo et al., 2003
**Lamiaceae**			
*Coleus aromaticus*	β-caryophyllene, bergamotene, and terpinolene	*Aedes aegypti*	Jaenson et al., 2006
*Hyptis martiusii*			
*Hyptis suaveolens*			
*Lavandula gibsoni*	α-Terpinolen and thymol	*Aedes aegypti, Anopheles stephensi*	Kulkarni et al., 2013
		*Culex quinquefasciatus.*	
*Lavandula stoechas*	Fenchone, 1,8-Cineole	*Culex pipiens*	Traboulsi et al., 2002
*Lippia origanoides*	Carvacrol	*Aedes aegypti*	Mar et al., 2018
*Mentha longifolia*	Piperitenone oxid	*Aedes aegypti*	Pavela et al., 2014
*M. microcorphylla*	Piperitenone, Pulegone, Piperitenone oxide	*Culex pipiens*	Traboulsi et al., 2002
*M. spicata*	Carvone	*Aedes aegypti*	Govindarajan et al., 2012
*Nepeta cataria*	E,Z-Nepetalactone and Z, E-nepetalactone	*Aedes aegypti*	Zhu et al., 2006
*Ocimum americanum*	E-Methyl-cinnamate	*Aedes aegypti*	Cavalcanti et al., 2004
*Ocimum basilicum*	Linalool; methyl eugenol	*Aedes aegypti*	Govindarajan et al., 2013
*Ocimum gratissimum*	Eugenol	*Aedes aegypti*	Cavalcanti et al., 2004
*Ocimum sanctum*	Methyleugenol	*Culex pipiens*	Gbolade and Lockwood, 2008
*O. syriacum*	Carvacrol, Thymol	*Aedes aegypti*	Traboulsi et al., 2002
*Perilla frutescens*	oleic, S-limonene, perillaldehyde	*Aedes aegypti*	Pohlit et al., 2011
*Plectranthus amboinicus*	Carvacrol	*Aedes aegypti*	Lima et al., 2011
*Plectranthus mollis*	Piperitone oxide, fenchone	*Aedes aegypti*	Kulkarni et al., 2013
*Pogostemon cablin*	Patchouli alcohol, Seyshellene, α-bulnesene, Norpatchoulenol	*Aedes aegypti*	Lima-Santos et al., 2019
*Pulegium vulgare*	Pulegone; carvone	*Aedes albopictus*	Pavela, 2015
*Rosmarinus officinalis*	1,8-Cineole; camphor	*Aedes aegypti*	Giatropoulos et al., 2018
*Satureja hortensis*	γ-Terpinene; carvacrol	*Culex pipiens*	Pavela, 2009
*Thymus capitatus (L.)*	Thymol, alpha-Amyrin, Carvacrol + beta-		Mansour et al., 2000
*Hoffm. & Link*	Caryophyllene	*Culex pipiens*	
*Thymus leucospermus*	p-Cymene	*Culex pipiens*	Pitarokili et al., 2011
*Thymus satureoides*	Thymol; borneol	*Culex pipiens*	Pavela, 2009
*Thymus teucrioides*	p-Cymene; γ-terpinene; thymol	*Aedes albopictus*	Pitarokili et al., 2011
*Thymus vulgaris*	a-terpinene,carvacrol, thymol		Giatropoulos et al., 2018
	p-cymene, linalool, geraniol	*Aedes aegypti*	
*Vitex agnus castus*	Trans-caryophyllene; 1,8 cineole	*Culex quinquefasciatus*	Niroumand et al., 2018
*Vitex trifolia*	Methyl-p-hydroxybenzoate	*Aedes aegypti*	Kannathasan et al., 2011
**Lauraceae**			
*Cinnamomum camphora*	1,8-Cineole	*Anopheles sinensis*	Zhang et al., 2018
*C. cassia*	Cinnamaldehyde	*Aedes aegypti*	Zhu et al., 2006
*C. impressicostatum*	Benzyl benzoate and α-phellandrene	*Aedes aegypti*	Jantan et al., 2005
*C. japonicum*	Borneol	*Anopheles sinensis*	Zhang et al., 2018
*C. microphyllum*	Benzyl benzoate	*Aedes aegypti*	Jantan et al., 2005
*C. mollissimum*	Benzyl benzoate	*Aedes aegypti*	Jantan et al., 2005
*C. osmophloeum*	*trans*-Cinnamaldehyde and cinnamyl acetate	*Aedes aegypti*	Cheng et al., 2004
*C. pubescens*	Benzyl benzoate	*Aedes aegypti*	Jantan et al., 2005
*C. rhyncophyllum*	Benzyl benzoate	*Aedes aegypti*	Jantan et al., 2005
*C. scortechinii*	β-Phellandrene and linalool	*Aedes aegypti*	Jantan et al., 2005
*C. sintoc*	Safrole	*Aedes aegypti*	Jantan et al., 2005
*C. subavenium*	Eugenol	*Anopheles sinensis*	Zhang et al., 2018
*C. szechuanense*	1,8-Cineole	*Anopheles sinensis*	Zhang et al., 2018
*Laurus nobilis*	1,8-cineole, linalool	*Culex pipiens*	Patrakar et al., 2012
*Lindera obtusiloba*	α-Copaene; β-caryophyllene	*Aedes aegypti*	Pavela, 2015
**Magnoliaceae**			
*Magnolia salicifolia*	Trans-anethole, Methyl eugenol, isomethyl eugenol, Costunolide, lactone and parthenolide	*Aedes aegypti*	Kelm et al., 1997
**Malvaceae**			
*Abutilon indicum*	β-sitosterol	*Aedes aegypti*,	Rahuman et al., 2008a
	Azadirachtin, salannin, deacetylgedunin, gedunin, 17-hydroxyazadiradione and deaceytlnimbin		
**Meliaceae**			
*Azadirachta indica*	Saponins	*Anopheles stephensi*,	Senthil-Nathan et al., 2005a
	23-O-methylnimocinolide		
	6α-O-acetyl-7-deacetylnimocinol	*Culex quinquefasciatus*	Ansari et al., 2005
	Nimocinolide; 7-O-deacetyl-23-O-methyl-	*Aedes aegypti*	Siddiqui et al., 1999
	7α-O-senecioylnimocinolide		Banerji and Nigam, 1984
	desfurano-6α-hydroxyazadiradione		Naqvi, 1987
	22,23-dihydronimocinol	*Aedes aegypti*	Siddiqui et al., 2002
	1α-acetyl-3α-propionylvilasinin	*Aedes aegypti*	Siddiqui et al., 2003
	Meliatetraolenone	*Aedes aegypti*	Siddiqui et al., 2003
	azadirachtin, salannin, deacetylgedunin,	*Culex quinquefasciatus*	Siddiqui et al., 2003
	gedunin, 17- hydroxyazadiradione		
	deacetylnimbin	*Anopheles stephensi*	Senthil-Nathan et al., 2005a
	3β,24,25-trihydroxycycloartane	*Anopheles stephensi*	
*Dysoxylum malabaricum*	Beddomei lactone	*Aedes aegypti*	Senthil-Nathan et al., 2009
*D. beddomei*	Caryophyllene epoxide	*Aedes aegypti*	Senthil-Nathan et al., 2009
	cis-Caryophyllene		
*Guarea humaitensis*	1α,7α,11β-triacetoxy-4α-carbomethoxy-	*Aedes aegypti*	Magalhães et al., 2010
*G. scabra*	12α-(2-methylpropanoyloxy)-14β,15β-epoxyhavanensin	*Aedes aegypti*	Magalhães et al., 2010
*Turraea floribunda*	1α,11β-diacetoxy-4α-carbomethoxy-7α-	*Aedes aegypti*	Ndung’u et al., 2004
	hydroxy-12α-(2-methylpropanoyloxy)-15-	*Aedes aegypti*	Ndung’u et al., 2004
	oxohavanensin; 1α-acetyl-3α-	*Culex pipiens*	Ndung’u et al., 2004
	propionylvilasinin	*Culex pipiens*	Ndung’u et al., 2003
*Turraea wakefieldii*	11β,12α-diacetoxyneotecleanin	*Culex pipiens*	Ndung’u et al., 2003
	11β,12α-diacetoxy-14β,15β-	*Aedes aegypti*	Ndung’u et al., 2003
	epoxyneotecleanin	*Aedes aegypti*	Ndung’u et al., 2003
**Myrtaceae**			
*Eucalyptus benthamii*	α-Pinene	*Aedes aegypti*	Lucia et al., 2012
*E. botryoides*	p-Cymene, α-eudesmol, and 1,8-cineol	*Aedes aegypti*	Lucia et al., 2012
*E. camaldulensis*	1,8-Cineol, p-cymene and β-phellandrene	*Aedes aegypti*	Lucia et al., 2008
*E. citriodora*	Citronellal; citronellol;	*Aedes aegypti*	Vera et al., 2014
	α-humulene isopulegol		
*E. dunnii*	1,8-Cineol and γ-terpinene	*Aedes aegypti*	Lucia et al., 2008
*E. fastigata*	p-Cymene	*Aedes aegypti*	Lucia et al., 2012
*E. globulus*	1,8-Cineol	*Aedes aegypti Anopheles arabiensis*	Massebo et al., 2009
*E. grandis*	α-Pinene	*Aedes aegypti*	Lucia et al., 2007
*E. gunnii*	1,8-Cineol and p-cymene	*Aedes aegypti*	Lucia et al., 2008
*E. nobilis*	1,8-Cineol	*Aedes aegypti*	Lucia et al., 2012
*E. radiata*	1,8-Cineol	*Aedes aegypti*	Lucia et al., 2012
*E. robusta*	α-Pinene	*Aedes aegypti*	Lucia et al., 2012
*E. saligna*	1,8-Cineol and p-cymene	*Aedes aegypti*	Lucia et al., 2008
*E. tereticornis*	β-Phellandrene and 1,8-cineol	*Aedes aegypti*	Lucia et al., 2008
*E. urophylla*	1,8-Cineol	*Aedes aegypti*	Cheng et al., 2009b
*E. melanadenia*	1,8-Cineol	*Aedes aegypti*	Aguilera et al., 2003
*Myrtus communis*	1,8 Cineole, α-Pinene, Linalool	*Culex quinquefasciatus*	Traboulsi et al., 2002
*M. dissitiflora*	Terpinen-4-ol	*Aedes aegypti*	Park et al., 2011
*M. leucadendron*	1,8-Cineol, α-pinene, and α-terpineol	*Aedes aegypti*	Leyva et al., 2008
*M. linariifolia*	Terpinem-4-ol and γ-terpinene	*Aedes aegypti*	Park et al., 2011
*M. quinquenervia*	1,8-Cineol and E-nerolidol	*Aedes aegypti*	Park et al., 2011
*Pimenta dioica*	Eugenol, linalool	*Aedes aegypti*	Pereira et al., 2014
*P. racemosa*	Terpinem-4-ol and 1,8-cineol	*Aedes aegypti*	Aciole, 2009
*P. guajava*	1,8-Cineol and β-caryophyllene	*Culex pipiens*	Leyva et al., 2009a
	1,8-Cineol	*Aedes aegypti*	Lima et al., 2011
*P. rotundatum*	Eugenol	*Aedes aegypti*	Aguilera et al., 2003
*Syzygium aromaticum*	Eugenol	*Aedes aegypti*	Costa et al., 2005
**Orchidaceae**			
*Vanilla fragrans*	4-ethoxymethylphenol, 4-butoxymethylphenol, vanillin, 4-hydroxy-2-methoxycinnamaldehyde and 3,4-dihydroxyphenylacetic acid	*Culex pipiens*	Sun et al., 2001
**Pinaceae**			
*Cupressus L.*,	limonene, α & β-pinene,	*Aedes aegypti*	Burfield, 2000
*Juniperus L.*	3-carene	*Aedes aegypti*	Burfield, 2000
*Pinus brutia*	α-Pinene and β-pinene	*Aedes albopictus*	Koutsaviti et al., 2015
*P. halepensis*	β-Caryophyllene	*Aedes albopictus*	Koutsaviti et al., 2015
*P. kesiya*	α-Pinene, β-pinene, myrcene and germacrene D.	*Aedes aegypti, Culex quinquefasciatus*,	Govindarajan et al., 2016
		*Anopheles stephensi*	
*P. longifolia*	k-terpineol	*Culex quinquefasciatus, Anopheles*	Ansari et al., 2005
		*culicifacies*	
*P. stankewiczii*	Germacrene D α-Pinene and β-pinene	*Aedes albopictus*	Koutsaviti et al., 2015
*P. sylvestris*	Eugenol 3, Cyclohexene-1-methanol, α-4-	*Aedes aegypti, Culex quinquefasciatus*	Fayemiwo et al., 2014
	trimethyl		
***Piperaceae***			
*Piper auritum* *P. betle* *P. capense* *P. decurrens*	Safrole Citronellal 2,3-Dihydro-2-(4′-hydroxyphenyl)-3-methyl-5(*E*)-propenylbenzofuran (conocarpan), 2-(4′-hydroxy-3′-methoxyphenyl)-3-methyl-5(*E*)-propenylbenzofuran (eupomatenoid-5), 2-(4′-hydroxyphenyl)-3-methyl-5(*E*)-propenylbenzofuran (eupomatenoid-6), 2,3-dihydro-5-formyl-2-(4′-hydroxyphenyl)-3-methylbenzofuran (decurrenal), and 3,7,11,15-tetramethyl-2(*E*)-hexadecen-1-ol (*trans*-phytol)	*Aedes aegypti* *Aedes aegypti* *Aedes atropalpus* *Aedes aegypti*	Leyva et al., 2009b Wahyuni, 2012 Chauret et al., 1996 de Morais et al., 2007
*P. gaudichaudianum*	Caryophyllene oxide, β-selinene	*Aedes aegypti*	de Morais et al., 2007
*P. hostmanianum*	Asaricin and myristicin	*Aedes aegypti*	de Morais et al., 2007
*P. humaytanum*	β-selinene, caryophyllene oxide	*Aedes aegypti*	de Morais et al., 2007
*P. klotzschianum*	1-Butyl-3,4-methylenedioxybenzene,	*Aedes aegypti*	do Nascimento et al., 2013
*P. longum*	limonene, and α-phellandrene	*Culex pipiens*	Lee, 2000
	Pipernonaline	*Aedes aegypti*	Yang et al., 2002
		*Aedes aegypti*	Costa et al., 2004
*P. marginatum*	Isoelemecin, apiole	*Aedes aegypti*	Autran et al., 2009
	(Z)-Asarone	*Aedes aegypti*	Autran et al., 2009
*P. permucronatum*	(E)-Asarone, patchouli alcohol	*Aedes aegypti*	de Morais et al., 2007
	Dillapiole and myristicin		
**Plumbaginaceae**			
*Plumbago zeylanica*	Plumbagin	*Aedes aegypti*	Pradeepa et al., 2016
**Poaceae**			
*Cymbopogon citratus*	Geranial	*Aedes aegypti*	Cavalcanti et al., 2004
*Cymbopogon flexuosus*	citral a-pinene	*Aedes aegypti*	Syed and Leal, 2008
*Cymbopogon nardus*	Geranial; neral	*Aedes aegypti*	Vera et al., 2014
	Girgensohnine	*Aedes aegypti*	Carreño-Otero et al., 2018
*Vetiveria zizanioides*	Citronellal	*Aedes aegypti*	Fradin and Day, 2002
	khusimol, isonootkatool, β-vetivenene, α &	*Aedes aegypti*	Vera et al., 2014
	β-vetivones		
**Papilonaceae**			
*Neorautanenia mitis*	Neotenone, neorautanone, pterocarpans neoduline, nepseudin,4-methoxyneoduline	*Culex quinquefasciatus, Anopheles gambiae*	Joseph et al., 2004
		*Aedes aegypti, Aedes albopictus*	
	Elemol, Eudesmols	*Culex quinquefasciatus*	Zhu et al., 2006
**Rutaceae**			
*Chloroxylon swietenia*	Heptacosanoic acid	*Aedes aegypti, Culex quinquefasciatus*	Balasubramani et al., 2015
*Citrus aurantifolia*	*Geijerene*, Limonene, Germacrene D	*Aedes aegypti, Anopheles stephensi*	Kiran et al., 2006
*Citrus hystrix*	α-terpineol		
*Citrus limon*	β-Pinene; d-limonene; terpinene-4-ol	*Culex pipiens*	Sutthanont et al., 2010
	Limonene	*Aedes aegypti*	
*Citrus reticulata*	D-Limonene; γ-terpinene	*Culex quinquefasciatus*	Michaelakis et al., 2009
*Citrus sinensis*	Limonene	*Aedes aegypti*	Sutthanont et al., 2010
	Limonin, Nomilin, Obacunone	*Culex quinquefasciatus*	Jayaprakasha et al., 1997
	Geijerene; limonene; germacrene D	*Aedes aegypti*	Vera et al., 2014
*Chloroxylon swietenia*			
			Kiran et al., 2006
*Clausena excavate*	Safrole and terpinolene	*Aedes aegypti, Aedes albopictus*	Cheng et al., 2009a
*Feronia limonia*	Estragole and β-pinene	*Aedes aegypti*	Senthilkumar et al., 2013
*F. limonia*	n-hexadecanoic acid	*Culex quinquefasciatus*	Rahuman et al., 2000
*Ruta graveolens*	Undecan-2-one	*Aedes aegypti*	Tabanca et al., 2012b
*Swinglea glutinosa*	β-Pinene; piperitenone;	*Aedes aegypti*	Vera et al., 2014
	α-Pinene		
*Toddalia asiatica*	Linalool	*Aedes aegypti*	Nyahanga et al., 2010
*Zanthoxylum armatum*	Linalool	*Aedes aegypti*	Tiwary et al., 2007
*Z. articulatum*	Viridiflorol	*Aedes aegypti*	Feitosa et al., 2007
*Z. avicennae*	1,8-Cineole	*Aedes albopictus*	Liu et al., 2014b
	Limonene	*Aedes aegypti*	Pitasawat et al., 2007
	Methyl heptyl ketone	*Aedes aegypti*	Borah et al., 2012
*Z. piperitum*	Asarinin, sesamin and (+)-xanthoxylol-γ,γ-dimethylallylether	*Aedes aegypti, Culex pipiens*	Kim and Ahn, 2017
*Z. monophyllum*	Germacrene D-4-ol and a-Cadinol	*Aedes albopictus, Culex quinquefasciatus, Anopheles stephensi*	Pavela and Govindarajan, 2017
***Santalaceae***			
*Santalum L.* spp.	α-santalol	*Aedes aegypti, Culex pipiens*	Jones et al., 2007
*Santalum album*	Guaiol, elemol, and eudesmol	*Anopheles stephensi*,	Amer and Mehlhorn, 2006
**Schisandraceae**		*Aedes aegypti*	
*Illicium verum*	Eugenol, α-Terpinyl acetate, Eucalypt, ol, (E)-anethole	*Culex quinquefasciatus*	Kimbaris et al., 2012
**Scrophulariaceae**			
*Capraria biflora L.*	α-Humulene	*Aedes aegypti*	Souza et al., 2012
*Stemodia maritima*	β-Caryophyllene and caryophyllene oxide	*Aedes aegypti*	Arriaga et al., 2007
**Tiliaceae**			
*Microcos paniculata*	*N-*Methyl-6b-(deca-l’,3’,5’-trienyl)-3b-methoxy-2bmethylpiperidine	*Aedes aegypti*	Bandara et al., 2000
**Verbenaceae**			
*Duranta repens*	β-amyrin and 12-oleanene 3β, 21β-diol,	*Culex quinquefasciatus*	Nikkon et al., 2010
*Lantana camara*	Bicyclogermacrene and E-caryophyllene	*Aedes aegypti*	Costa et al., 2010
	Eucalyptol, caryophyllene,		
*Lippia alba*	Carvone; limonene	*Aedes aegypti*	Santiago et al., 2006
*L. gracilis*	Carvacrol	*Aedes aegypti*	Santiago et al., 2006
*L. origanoides*	Carvacrol; p-cymene	*Aedes aegypti*	Vera et al., 2014
*L. javanica*	Allopurinol,camphor, Limonene, a –terpeneol, verbenone	*Aedes aegypti*	Mwangi et al., 1992
*L. microphylla*	1,8-cineole, thymol, α-pinene	*Aedes aegypti*	Santiago et al., 2006
*L. nodiflora*	Camphor, *p*-cymene, γ−terpinene	*Aedes aegypti*	Santiago et al., 2006
*L. sidoides*	Thymol	*Aedes aegypti*	Costa et al., 2005
**Zingiberaceae**			
*Alpinia purpurata*	β-Caryophyllene and β-pinene	*Aedes aegypti*	Santos et al., 2012
*Curcuma aromatic*	1H-3a,7-Methanoazulene and curcumene	*Aedes aegypti*	Choochote et al., 2005
	Turmerone, curcumene, and zingiberene		
*Curcuma longa*	1,8-Cineol and p-cymene	*Aedes aegypti*	Leyva et al., 2008
*Curcuma zedoaria*	Dodecanal	*Aedes aegypti*	Pitasawat et al., 2007
*Hedychium coccineum*	1,8-Cineol and β-pinene	*Aedes aegypti*	Sakhanokho et al., 2013
*Hedychium* sp.	1,8-Cineol	*Aedes aegypti*	Sakhanokho et al., 2013
*Kaempferia galanga*	Ethyl trans-p-methoxycinnamate	*Aedes aegypti*	Munda et al., 2018
*Kaempferia galanga*	Ethyl cinnamate	*Aedes aegypti*	Munda et al., 2018
*Zingiber officinale*	4-Gingero	*Aedes aegypti, Culex quinquefasciatus*	Rahuman et al., 2008b
*Zingiber officinale*	6-Dehydrogingerdione	*Aedes aegypti, Culex quinquefasciatus*	Rahuman et al., 2008b
*Zingiber officinale*	6-Dihydrogingerdione	*Aedes aegypti, Culex quinquefasciatus*	Rahuman et al., 2008b
*Zingiber zerumbet*	α-Humulene; zerumbone	*Aedes aegypti*	Sutthanont et al., 2010

Besides secondary metabolites, essential oils (EOs) from plants were also recorded with effective mosquitocidal potentials. The EOs from the plants of *Lamiaceae* and *Zingiberaceae* were proved with bioactivity against *Ae. aegypti* (Kalaivani et al., 2012). The fern *Actiniopteris radiata* was testified with novel mosquitocidal activity against larvae of *Ae. aegypti* and *An. Stephensi* (Kamaraj et al., 2018). The seed oil extract of *Acacia nilotica* possessed robust larvicidal action against major mosquito vectors (Vivekanandhan et al., 2018a). A remarkable biological activity of EOs against Dengue vectors has been extensively reviewed by Chellappandian et al. (2017, 2018, 2019). Plant volatile oils were also conveyed with mosquitocidal potentials. As studied by Vasantha-Srinivasan et al. (2018), the crude volatile oil (CVO) from Piper beetle leaves possessed significant larvicidal, ovipositional, and repellency effects against *Ae. Aegypti*.

Derivatives of plants are enriched with active molecules with exceptional mosquitocidal properties and can be advanced as low cost environmentally friendly bio-pesticides. Many botanical extracts along with their chief constituents showed effective insect metabolism inhibition or stimulation of digestive enzymes (Senthil-Nathan et al., 2009; Napoleão et al., 2012; Senthil-Nathan, 2013). Unlike synthetic chemicals, previous literature on plant compounds doesn’t provide any indication for the emergence of resistance so far. This is most likely due to the blend of several bioactive compounds with different mechanisms of action and therefore it is difficult for mosquito vectors to develop resistance (Mulla and Su, 1999; Shaalan et al., 2005).

## Impact of Phytochemicals on the Physiology of Mosquito Larvae

As in general, plant secondary metabolites are evolved as protection mechanism against herbivory. When these toxic substances are encountered by the mosquitoes, a relatively unambiguous response is triggered that has a non-specific influence on a wide range of molecular targets such as proteins, nucleic-acids, bio-membranes, besides added cellular components. Consequently, the physiology is disrupted at numerous receptor sites, eventually causing an abnormality in the nervous system. Plant metabolites affect several vital physiological functions that include inhibition of “AChE” as well as “GABA-gated” chloride channel, disruption of Na–K ion exchange besides constricting the cellular respiration. As a subsequent event, the alteration of these enzyme levels gives rise to several anomalies that include the obstruction of nerve cell membranes and octopamine receptors along with calcium channel blockage, resulting in hormonal imbalance, mitotic poisoning, and also modifications of the molecular basis of morphogenesis (Rattan, 2010).

**FIGURE 1:**
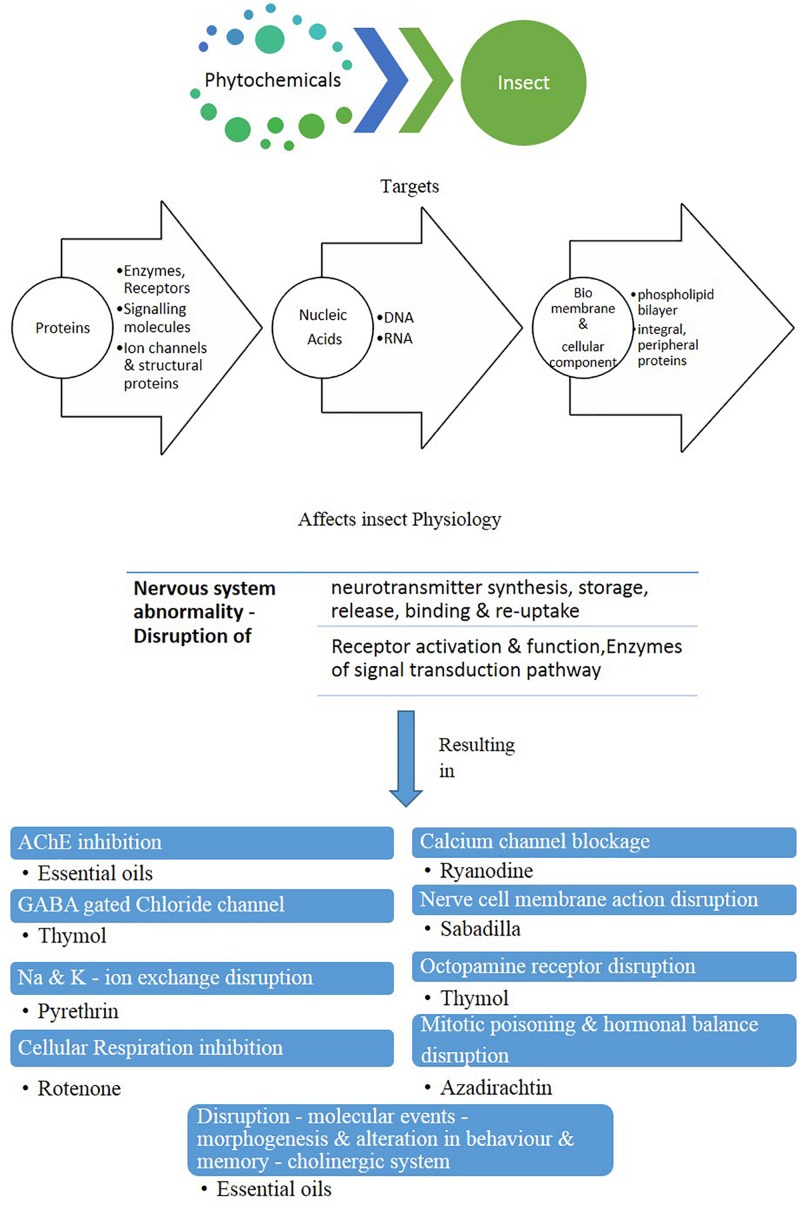
Mode of action of phytochemicals in insect body (modified after Ghosh et al., 2012).

Synthetic insecticides generally increase the level of detoxifying enzymes. Phytochemicals target the mentioned cellular mechanisms and potentially disturb their functions (Figure 1; Zibaee and Bandani, 2010; Zibaee, 2011; Kaur et al., 2014; Senthil-Nathan, 2015). Physiological effects of phytochemicals are discussed below.

## Impact of Phytochemicals on Detoxifying Enzymes

The antioxidant and detoxification enzymes of mosquito vectors are vital in detoxification of reactive oxygen species (ROS) synthesized by the toxic chemicals (Rattan, 2010). Esterase and phosphatase of the mosquito vectors plays a key role in several physiological events (Koodalingam et al., 2014). Excessive usage of toxic chemicals on mosquito control caused insecticide resistance through sodium channel mutations, activation of detoxification enzymes, and upregulation of key genes and other regulatory components like MicroRNAs (miRNAs). The CYP450s, GSTs, SOD, and esterase gene families are recognized as the foremost four enzymes accountable for the metabolic-resistance of the insects (Hemingway et al., 2004). Generally, detoxifying enzymes are involved in digestion, reproduction, juvenile hormone metabolism, neuronal conduction, moulting, and more importantly detoxification of toxic chemicals (Koodalingam et al., 2014). Phosphatases are involved in tissue development, cellular differentiation, carbohydrate metabolisms, and synthesis of ATP (Koodalingam et al., 2014). Mainly these two major classes of detoxifying enzymes are considered for evaluating the impact of toxic chemicals on physiological or biochemical events of arthropod vectors.

Carboxyl-esterases (EC3.1.1.1) are non-specific omnipresent enzymes that are associated to the major “endogenous” functions in insects, which hydrolyze a different carboxylic-acid ester (Lija-Escaline et al., 2015). Generally, the metabolic pathway of these enzymes was targeted by the chemical pesticides, especially the fourth generation class of Pyrethroids, which acts on the voltage sensitive sodium channels and blocks the mosquito nervous system (Hong et al., 2014). Esterases can also target by sequestering the insecticide through rapid binding and slowly releasing the insecticide metabolites (Karunaratne et al., 1993). This latter type of resistance requires the presence of increased quantities of esterase due to the 1:1 stoichiometry of the reaction and decreases the metabolic breakdown time.

Plant extracts and their derivatives have been widely reported to decrease the levels of carboxylesterase (α- β-carboxylesterase) level in the *Ae. aegypti* larva (Koodalingam et al., 2014; Lija-Escaline et al., 2015). Besides exhibiting larvicidal activity *Alangium salvifolium*, also substantially reduced the levels of α, β-carboxylesterase as well as superoxide dismutase (SOD) in *Ae. Aegypti* (Thanigaivel et al., 2017a). *Myrrh commiphora molmol* (oil and oleo-resin extract) instigated biochemical changes in *Cx. pipiens* that affected the cell proteins, as well as loss of enzyme activity (Massoud et al., 2001).

Higher rates of enzyme activities, such as SOD (Agra-Neto et al., 2014; Lija-Escaline et al., 2015) and physiological enzymes like esterase (Wheelock et al., 2005; Lija-Escaline et al., 2015), phosphatases (Walter and Schütt, 1974; Urich, 1994) are recorded with increasing developmental stages and these are considered responsible for increased pyrethroid resistance. The Mosquito vectors that established resistance to Temephos have been found to possess genes that insensitized ACHE on exposure to pesticides. Insects were also characterized by the over expression of varied forms of detoxifying enzymes (GST, SOD, and esterases) (Larson et al., 2010).

Glutathione-*S*-transferases are a class of detoxification enzymes considered to play a vital role in the existence of insects exposed to toxic metabolites. Increased GST activities are connected with the expression of metabolic resistance toward insecticides (Clark, 1990). GSTs can break down a broad range of substances; amplified GST activity is possibly as a response to an environmental stress. Generally, Cytochrome P450s (CYP450) displayed upregulation when induced by plant secondary metabolites in diverse insect pests especially against the vectors of human diseases (Caballero et al., 2008) and have members which are considered as major elements conferring resistance against insecticides (i.e., CYP2, CYP4, and CYP6) (Sun et al., 2001). The upregulation of GST enzymes usually at the exposure of a prominent dosage of plant compounds suggests the activity of a major detoxification process (Edwin et al., 2016). Consequently, the levels of GST expression may be used as a biomarker to detect the development of resistance (Jukic et al., 2007).

CYP450 group of enzyme family are also designated as key indicators of metabolic resistance besides susceptibility to insecticides (David et al., 2013). Many previous research outcomes proved alteration or inhibition in the expression of major detoxifying enzymes exposed to plant chemicals. Thanigaivel et al. (2017a) showed increase in the rate of GST activity in IV instar larvae of dengue mosquito exposed to methanolic leaf extract of *J. adhatoda* with their major derivative 3-hydroxy-2,3-dihydropyrrolo[2,1-b]quinazolin-9(1h) one (26.37%). Likewise, carboxylesterase activities differed significantly in *Ae. aegypti* post treatment with the leaf extracts of *P. nigrum* with their major derivatives thymol (20.77%) (Lija-Escaline et al., 2015). Correspondingly, the activity of major enzymes (esterases, GST, and CYP450) of dengue mosquito severely affected post treated with dynamic plant compound andrographolide derived from *Andrographis paniculata* (Acanthaceae) at the maximum dosage of 12 ppm (Edwin et al., 2016). DDT resistance in the mosquito *An. gambiae* is correlated elevated glutathione transferase (GST) E2 activity (*Ag*GSTE2) (Enayati et al., 2005). The DDT resistant *An. gambiae* evades the insecticidal activity by the dehydrochlorination of DDT to its non-insecticidal metabolite DDE. Muleya et al. (2008) reported that compounds -epiphyllocoumarin (Tral-1), knipholone anthrone, isofuranonaphthoquinones (Mr 13/2, Mr13/4), and the polyprenylated benzophenone (GG1) were potent inhibitors of AgGSTE2.

Besides the botanical extracts, EO derived from the plants also have strong inhibition of detoxifying enzymes of arthropod vectors (Pavela, 2015). EOs may provide substitute sources of vector control since they are enriched with diverse phyto-molecules with insecticidal properties (Cheng et al., 2013). Insecticide phytochemicals from EOs belong to terpenoids chiefly and Phenylpropanoids to a limited extent. In which, Terpenoids includes monoterpenes and sesquiterpenes as the major compositions of EOs (Chellappandian et al., 2018). Lee et al. (2003) specified that volatile and lipophilic monoterpenoids infiltrate insect body, where they afflict physiological processes, and hence their mode of action is hard to elucidate. Previous research of Vasantha-Srinivasan et al. (2017) showed that the CVO derived from *Piper betle* (L.) (Pb-CVO) showed upregulation in the level GST and CYP450 and down regulate the expression of Carboxylesterases activity against the field and laboratory strains of *Ae. aegypti*. Moreover, the above results also showed that the changes in the level of enzymes are steady in both field and laboratory strains compared to the chemical pesticides. Due to enriched chemical diversity and potential mosquitocidal activity, CVO have acquired greater interest from researchers looking for new besides natural replacements to chemical-pesticides in controlling medically challenging pests (Pavela, 2015). Correspondingly, EO constituent’s nootkatone and carvacrol from Alaskan yellow cedar tree inhibits 50% of acetylcholine esterase activity in *Ae. aegypti* compared to the carbaryl, a known acetylcholinesterase inhibitor (Anderson and Coats, 2012). The impact of major plant molecules against the mosquito larvicides was tabulated (Table 1). Hence, expression of these molecules on detoxifying and metabolic enzymes is considered an important biomarker to evaluate the mosquitocidal potential of bio-rational plant metabolites.

Pradeepa et al. (2014) have reported the antimalarial activities from the compound plumbagin, identified from the rhizome of *Plumbago zeylanica* against *An. stephensi*. Also, it was revealed that plumbagin constrains the vector AchE enzyme, *An. Stephensi* in a dose dependent manner and also can be considered for controlling resistant vectors whose insecticide resistance is associated to an increased SOD activity (Pradeepa et al., 2016). The detection of SOD activity in the anal gills of *An. stephensi* larvae could be associated with their resistance provided against damaging oxygen products (Nivsarkar et al., 1991). The sensitivity of an insect to an insecticide can hence be increased by identifying certain compounds that can deactivate these enzymes (Larson et al., 2010).

## Impact of Phytochemicals on Midgut Tissues

The midgut of the mosquito larvae is the chief interface of exterior environment and chip in major process like digestion, ion transport, absorption, and osmoregulation process (Bernick et al., 2008; Elumalai et al., 2016). Generally, gut region is the target of numerous insecticidal complexes and its integrity is dynamic for digestion and conferring of resistance against toxins (Stenfors Arnesen et al., 2008). With the insect midgut being the important site for synthesis of digestive enzymes, plant derived molecules primarily targets thee gut epithelium layer (EL) (Senthil-Nathan et al., 2008). This might be the significant cause for condensed metabolic rate in addition to a reduced enzyme activity (Selin-Rani et al., 2016). The peritrophic membrane (pM) gaurds the EL from the surrounding the gut lumen (GL) (Lija-Escaline et al., 2015). Phyto-chemicals are proven to exert a serious impact on the digestive epithelial cells and further decrease the growth rate of arthropods (Yu et al., 2015). Neira-Oviedo et al. (2008) stated that plant compounds flow into the gastric caeca and the malpighian tubules thereby affecting the midgut epithelium. For instance, extracts of *M. azedarach* have been reported to cause extensive harm on the EL and pM of filarial vector *Cx. quinquefasciatus* (Al-Mehmadi and Al-Khalaf, 2010). The pM may influence the growth and development of parasites vectors by creating a mechanical barrier to invasion by ookinetes (Rudin and Hecker, 1989). Plant extracts and their metabolites are crucial for the impairment of pest mid-gut epithelium (Rey et al., 1999). The compound catechin isolated from *Leucas aspera* affects the mid-gut of the three mosquito larvae *Ae. aegypti*, *An. stephensi*, and *Cx. quinquefasciatus* (Elumalai et al., 2016). Previous photomicrographic study on the midgut tissues of the dengue mosquito (Field and laboratory strains of *Ae. aegypti*) treated with the CVO of *P. betle* displayed severe injuries to the GL and EL (Vasantha-Srinivasan et al., 2018). Correspondingly, leaf extracts of *Aristolochia indica* L. (*Aristolochiaceae*) and their derivatives aristolochic acid I and II showed severe damage on the midgut vacuolated gut epithelial columnar cells (epi), GL, and pM (Pradeepa et al., 2015). Likewise, methanolic leaf extracts of *P. nigrum* severely affected the midgut cellular organelles of *Ae. aegypti* at the minimal dosage of 10 ppm (Lija-Escaline et al., 2015). Similarly, Vasantha-Srinivasan et al. (2018) reported that *P. betle* CVO derived from *P. betle* at the sub-lethal dosage damage the pM, and major alteration in the alignment of EL and GL of dengue mosquito comparable to the control. Previous research on Andrographolide a major derivative of *A. paniculata* against dengue mosquito gut cells proved that there was an unembellished collapse in the mid-gut pM, in addition to a chief variation in the El and GL alignment (Edwin et al., 2016). Selin-Rani et al. (2016) reported that the active plant molecules may damage the gut epithelium is the vital reason for concentrated metabolic rate and decrease in the enzyme-activity. Midgut cell damage is directly linked to the digestive and detoxifying enzymes dysregulation (Senthil-Nathan et al., 2008). This was also confirmed by histological studies of the mosquitoes that displayed midgut cell damage, post treatment with various botanical compounds (Yu et al., 2015). Further, treatment with plant compounds were also associated with altered protein (Fallatah, 2014) and biochemical profiles in mosquitoes (Senthilkumar et al., 2013).

Biochemical studies on *Cx. pipipens* exposed to *Allium satvium*, *Citrus limon*, and Bti were observed by Saeed et al. (2010). Results revealed that the use of plant oil extracts and *Bti* have great effect on total protein content of treated mosquito larvae. Fallatah (2010) reported the effect of water extract of fenugreek have high larvicidal effect against *Cx. quinquefasciatus*, causing noticeable effects on numerous body tissues together with the midgut and nervous system as well as total protein content. Aristolochic acids isolated from *A. indica* Linn, mainly affected the midgut EL and secondly the larval muscles and cells (Pradeepa et al., 2015). Similar results were also observed in mosquitoes treated with plant extracts (Costa et al., 2012). The orientation of the cytoplasmic protrusions of the apical surfaces of columnar cells toward the lumen suggests the secretion of apocrine and/or apoptosis.

Al-Mekhlafi (2018) reported the effect of *Arum copticum* (*Apiaceae*) extract *against Culex pipiens* larvae. Apart from exhibiting larvicidal activity, the extract was able to display cytopathological alterations of the midgut epithelium. EO and enriched fraction of *Peumus boldus* displayed larvicidal activity against *Cu. Quinquefasciatus*. The treated larvae displayed morphological changes in the midgut cells (de Castro et al., 2016). Velu et al. (2015) tested the peel extract of *A. hypogaea* against *Aedes aegypti* and *Anopheles stephensi*. The histopathological studies exposed midgut tissue damage and cuticle injury. Costa et al. (2012) reported similar aberrations in *Ae. aegypti* larvae (III instar) treated with *Annona coriacea* extract. *Ae. aegypti* larvae exposed to squamocin from *Annona mucosa* Jacq. (Annonaceae) displayed larvicidal and cytotoxic action with changes in the midgut epithelium and digestive cells by increasing the expression of autophagy genes (Costa et al., 2014, 2017). da Silva Costa et al. (2018) also reported that squamocin affected the osmoregulation and ion-regulation of *Ae. aegypti* larvae which resulted in a lethal effect caused by the development of a great vacuolization in the anal papillae wall.

The histopathological study of *Ae. aegypti* treated with methanol extract derived from seaweeds *Sargassum binderi* showed that larvae treated with seaweed extracts had cytopathological alteration of the midgut epithelium. The morphological observation revealed that the anal papillae and terminal spiracles of larvae were the common sites of aberrations (Yu et al., 2015). Phytochemicals (oleic, linoleic, linolenic, palmitic, and stearic acids) and their respective methyl esters were tested against fourth instar *Cu. quinquefasciatus* larvae. The compounds were found to affect its metabolism and the morphology of midgut along with their fat body (de Melo et al., 2018).

## Impact of Phytochemicals on the Insect Behavior

With the development of resistance by this time attained to almost all available chemicals, strategies integrating “plant derived” compounds to influence “semiochemical”-mediated behaviors by means of interruption of mosquito-olfactory sensory system have substantially developed (Muema et al., 2017). As a consequence, the physiological status related to the olfactory sensory system is disrupted. The phytochemicals will bind to these odorant chemoreceptors and subsequent flight orientations of the mosquitoes are hindered (Bohbot et al., 2010). Henceforth the physiological status for instance “circadian-regulated appetitive stimulus” or “gonotrophic status” that triggers olfaction in pursuit of nutritious sources, mates and oviposition sites are disturbed. Plant-based semiochemicals can be exploited to lure the mosquitoes to an insecticide trap, thereby forming an integral part of an integrated vector control programe (Kamala-Jayanthi et al., 2015). Rice volatiles on evaluation with BioGent (BG) sentinel traps elicited antennal responses that stimulated long range oviposition site seeking behavior. Also, p-cresol, from Bermuda grass hay infusion was reported with avoidance response to gravid *An. Gambiae* (Eneh et al., 2016).

## Future Perspectives

Higher rates of anthropogenic activities that are expected to expand with the population increase will increase the incidence of vector borne diseases. Additionally, the development of resistance among the vector population against the synthetic chemical insecticides along with their persistence in the environment and toxicity for non-target organisms are reducing the efficiencies of vector management practices globally. Hence novel plant-based compounds that are safe and effective are being focused for the development of improved management of vectors.

The research has now moved on from the isolation of bioactive compounds with anti-vector potentials to formulate novel application methods. Apart from the direct application of plant metabolites in vector control, nanoparticles (NPs) synthesized from plants using green technology are emerging as a new trend. Nanotechnology is presently “revolutionizing” the manufacture of commercial pesticides. Production of green NPs and nanoencapsulation compounds upsurges the permanence of EOs through “slow-release” phenomenon deliberating sustained fortification against mosquito bites. As reported by Jinu et al. (2018), silver nanoparticles (AgNPs) from *Cleistanthus collinus* Karra and *Strychnos nux-vomica* Linn *nux-vomica* presented highest larvicidal activity against *A. stephensi* and *A. aegypti*. Murugan et al. (2018a, b) proved the efficacy of zinc oxide NPs fabricated using the brown macroalga *Sargassum wightii* Greville ex J. Agardh. against *An. stephensi.* In another study reported by Murugan et al. (2018b), Poly (Styrene Sulfonate)/Poly (allylamine hydrochloride) encapsulation of TiO_2_ NPs were found to enhance their toxicity against mosquito vectors of Zika virus.

## Conclusion

Mosquito vector borne diseases are a major human health problem in all countries. There has been an alteration toward plant-based insecticides to overcome the problems related with the use of synthetic mixtures in mosquito control programe. Botanicals can be used as mosquitocides for killing both larvae and adult mosquitoes. However, only very few botanicals have moved from laboratory to the field use, which may be due to the light and heat variability of phytochemicals compared to synthetic insecticides. Further these botanicals have been widely explored, but only a comparatively small number of patents have been filed with the persistence of regulating the formulations for use against mosquito species in the field level.

Although the activity of phytochemicals are generally attributed to some specific compounds, but there is increasing evidence that the combination of botanicals and biopesticides will result in an increased bioactivity compared to single phytochemicals (Senthil-Nathan et al., 2005a; Senthil-Nathan and Kalaivani, 2005, 2006).

At present, botanical insecticides make <1% of the world’s pesticide market (Sola et al., 2014). Isolation of active principles and synthesis of secondary metabolites of botanicals against mosquito threat are very important for the management of vector borne diseases. The positive results of initial studies on larvicidal potential of botanicals encourage further interest to investigate the bioactive compounds. Identifying botanical insecticides that are effective as well as appropriate and adaptive to overcome ecological hazards, biodegradable, and have a broad spectrum of larvicidal properties will work as a new defense in the arsenal of insecticides and it may act as an appropriate alternative product to fight against vector-borne diseases.

Thus, the present review collects important information on plant extracts along with their active molecules as agents affecting the physiology and behavior of medically threatening mosquito vectors. Now collective efforts are needed to take advantage of the accumulated knowledge on phytochemical action on mosquitos in order to integrate their application in integrated pest management programs.

## Author Contributions

SS-N collected all the information and wrote the review.

## Conflict of Interest

The author declares that the research was conducted in the absence of any commercial or financial relationships that could be construed as a potential conflict of interest.
